# Maximal Intensity Periods During International Male Field Hockey

**DOI:** 10.1002/ejsc.12333

**Published:** 2025-06-09

**Authors:** Paul S. R. Goods, Brendyn Appleby, Brendan R. Scott, Peter Peeling, Brook Galna

**Affiliations:** ^1^ PHysical Activity, Sport, and Exercise (PHASE) Research Group School of Allied Health (Exercise Science) Murdoch University Perth Australia; ^2^ Centre for Healthy Ageing Health Futures Institute Murdoch University Perth Australia; ^3^ Hockey Australia High Performance Program Perth Australia; ^4^ School of Human Sciences (Exercise and Sport Science) The University of Western Australia Perth Australia

**Keywords:** acceleration, movement, team sport, time‐motion, worst case scenario

## Abstract

In this retrospective cohort study, we examined maximal intensity periods (MIPs) for a broad range of movement characteristics during international field hockey. Further, we examined the intensity of near‐peak periods, and whether peak demands for different movement characteristics occurred simultaneously. Player movement data from 28 Australian elite male field hockey players were obtained via wearable tracking devices in four international tournaments over 13 months (*n* = 393 player‐matches). MIPs were identified via the rolling‐sum method for mean speed, high‐speed distance (> 5 m·s^−1^), accelerations (> 2.5 m·s^2^), decelerations (< − 2.5 m·s^2^) and high‐speed cuts (45° change of direction and > 5 m·s^−1^) across eight epochs (range: 5 s–5 min). Random effects linear mixed models were used to estimate means for each movement characteristic, with random intercepts fitted for players and matches. Mean speed was ∼80% higher during the 1 min MIP (210 m·min^−1^) than the match average (116 m·min^−1^) and players regularly reached high mean speeds (for instance, the 10th most intense minute was still ∼44% above match average). High‐speed distance, accelerations and decelerations accumulated > 5x faster during the 1 min MIP for those variables than the match average and high‐speed cuts occurred with ∼10x greater frequency. During the 1 min MIP for total distance, all other movement characteristics were less than 40% of the 1 min MIP for that variable (except high‐speed distance: 76%). Match averages substantially underestimate the MIPs of elite field hockey. Practitioners should consider analysing the peak periods of matches, with a focus on high‐intensity movements, to inform monitoring and prescription of team sport‐specific training.

**Summary**

- All maximal intensity periods investigated here (from < 1 to 5 min) are substantially more intense than mean match demands. Practitioners working in field hockey should consider analysing the peak periods of matches, with a focus on high‐intensity movements, to inform their monitoring and prescription of team sport‐specific training. Practitioners should consider both the duration of their training drills and the corresponding MIP of that duration in peak periods of matches.
- The most intense periods of the match for total distance (i.e., highest mean speeds) do not occur at the same time as the most intense periods for other high‐intensity movements such as accelerations, decelerations and changes of direction; therefore, practitioners should avoid using only mean speed to characterise field hockey‐specific drills, such as small‐sided games.
- Elite field hockey players regularly run at near maximal intensities throughout matches, so practitioners should ensure that they train and monitor this physical capability.

## 1 Introduction

The movement demands of team sport athletes have been studied for over 50 years (Reilly and Thomas 1976). For a long time, laborious methods were employed to report match data, such as total distance, often discretised into speed bands (Docherty et al. 1988), and eventually with special attention on high‐intensity movements during matches (Bangsbo et al. 1991). These data are intended to inform practitioners about normative values for different sports and populations, aiding talent identification, training monitoring and training prescription processes. However, with the proliferation of global navigation system technology in the 21st century, more sophisticated analytics have emerged. One such analysis is the maximal intensity period (MIP; Weaving et al. 2022), which quantifies the most intense discrete periods during team sport matches. It is suggested that analysis of MIPs could help practitioners prescribe and monitor the intensity of team sport specific training drills, which typically last from between several seconds to several minutes, to ensure that these drills are eliciting match‐like or greater intensities for commensurate durations (Weaving et al. 2022).

In field hockey, time‐motion analysis has also been reported for several decades (Lothian and Farrally 1994; Spencer et al. 2004), yet relatively little information is available on MIPs. The first MIP analysis in field hockey was performed on 28 highly trained athletes competing in an Australian national tournament, with speed traces trimmed to only include match time (Delves et al. 2021). Peak moving average values were determined for speed, acceleration and metabolic power for 1–10 min epochs, in 1 min bands, with mean speeds observed to be 50%–65% greater in the 1 min MIP than previously reported for whole‐match averages. In the first investigation of international‐level athletes, Cunniffe et al. (2021) examined differences in mean speed for 27 field hockey players across three competition levels (amateur, professional and international). However, only one variable (mean speed) and one MIP (3 min) was reported, with the unexpected finding that professional club matches elicited a greater MIP than amateur or international matches. The authors postulated that this was possibly due to the level of competition in the club (Irish Hockey League) and international data (several matches played against low‐ranked teams > 15th in the world) not meeting the level of competition of the professional leagues included in the analysis (Belgian, Dutch and German leagues, considered the strongest in the world). McGuinness et al. (2022) explored mean speed, high‐intensity running (> 16 km·h^−1^) and sprinting (> 20 km·h^−1^) for 1–10 min epochs, in 1 min bands in 23 international female field hockey players, also reporting greater values for all MIPs than previously observed for whole‐match averages. Most recently, Büchel et al. (2023) investigated an 8‐week in‐season training and competition cycle for 20 amateur field hockey players from the German first division, where they analysed mean speed, high‐intensity running (> 16 km·h^−1^), sprinting (> 20 km·h^−1^) and mean acceleration load for 1–5 min epochs, in 1 min bands. The key finding reported by Büchel et al. (2023) was that training failed to elicit intensities similar to peak periods of a match in any variable (time‐matched MIPs were 40%–60% greater in matches than training), which supports the rationale for implementing MIP analysis to help inform the training process.

Collectively, these investigations highlight the practical utility of MIP analysis, with all researchers consistently observing far greater movement demands during peak periods of the match compared to match averages, and Büchel et al. (2023) confirming that these intensities may not be replicated in training. However, with relatively little research in this area to date, several important questions remain unanswered or under‐investigated. For instance, only two investigations have reported data from international competition, with the authors of the only available international male data citing concerns with the level of opposition included in their analysis (Cunniffe et al. 2021). Further, this sole investigation of international male field hockey only examined a single variable (mean speed) and MIP (3 min), which means no information is available on high‐speed running, acceleration‐based or change of direction variables, which are commonly reported in field hockey investigations (Büchel et al. 2023; Delves et al. 2021; Goods, Scott, et al. 2023; Goods et al. 2024; McGuinness et al. 2022). Therefore, our primary aim was to examine the MIPs for a broad range of speed, acceleration‐based and change of direction variables during international male field hockey. In addition, we had three secondary aims: (1) to identify any variables that occur too infrequently to warrant MIP analysis, (2) to report the intensity of other intense periods of the match (which we will define as the next nine most intense periods and refer to as near MIPs throughout) and (3) to explore whether peak demands for different movement characteristics occurred simultaneously during the match.

## 2 Materials and Methods

We investigated the player movement data from 28 Australian elite and world class (i.e., tiers 4 and 5) (McKay et al. 2022) male field hockey players. Data were obtained across ∼13 months from four international tournaments (2023 Men's Fédération Internationale de Hockey [FIH] Hockey World Cup, 2023 Men's FIH Pro‐League, 2023 Oceania Cup and 2024 Men's FIH Pro‐League). A total of 25 official international matches were included in the analysis, with 21 of these matches (84%) played against opponents ranked top 10 in the world at the time of the match (range: 1–12 on the FIH Men's World Ranking, October 2024). A large sample of tournaments and matches across > 1 y was chosen to minimise the impact of context‐specific factors on the data (e.g., match location, match proximity to travel or start of tournament, environmental conditions or opponent ranking). Due to the nature of international field hockey tournaments, matches were often played on consecutive days or in a cluster of four to five matches with a single rest day.

Only field players were included in this analysis (i.e., goal keepers excluded). As the data collection window spanned > 1 y, players and their demographic information (e.g., age, body mass and fitness) varied for the different tournaments; however, all players represented Australia in at least one of the selected international tournaments. Participants consented (in writing) for their data to be used for research publication purposes as a part of their athlete agreement, with institutional ethical approval obtained prior to retrospectively analysing de‐identified player movement data (2021/ET000838).

All players wore the same wearable tracking device (Vector X7, Catapult Sports, Melbourne, Australia) between their scapulae in a manufacturer‐supplied sports vest for each match included in this analysis. Each device contains a global positioning system (GPS) and global navigation satellite system (GNSS), each with a sampling rate of 10 Hz. All player movement data were collected by the national team staff and extracted via a proprietary software (OpenField v3.3.1, Catapult Sports, Melbourne, Australia) prior to being bulk exported, deidentified and provided to the research team. Data were extracted using default export settings, and no additional filtering or smoothing was applied except when calculating change of direction. The GPS/GNSS technology in the Catapult Vector system has been demonstrated to be valid and reliable for both linear and multidirectional movement, with no decreases in validity or reliability as speed increases (Varley et al. 2023).

Player movement data were trimmed to exclude periods between quarters and where players were on the bench (i.e., including only ‘active duration’). ‘Total distance’ (m) was calculated as the two‐dimensional distance covered by players during active duration and was further discretised as the distance covered when travelling > 5 m·s^−1^ (‘high‐speed distance’) and > 7 m·s^−1^ (‘sprint distance’). ‘High‐intensity efforts’ were defined based on recent research in international field hockey (commencing with an acceleration of 2.5 m·s^2^ over 1 s and ceasing when speed dropped 1 m·s^−1^ below peak speed during the effort; Goods et al. 2024). Additionally, discrete ‘accelerations’ (> 2.5 m·s^2^) and ‘decelerations’ (< − 2.5 m·s^2^) were quantified using GPS/GNSS data, with a buffer of one second imposed between consecutive accelerations (or decelerations) to avoid counting the same acceleration or deceleration event multiple times. Finally, a ‘high‐speed cut’ was defined as an event when a player changed direction by at least 45° over one second and was moving faster than 5 m·s^−1^ at any stage within than second. To identify 45° changes of direction, raw coordinate data obtained from the GPS/GNSS device were imported into MATLAB (v2023b, Mathworks, USA) and Cartesian coordinates were calculated using the World Geodetic System of 1984 (WGS84) reference ellipsoid. Data were then filtered (3rd order, 1 Hz, low‐pass Butterworth filter) before reconstructed coordinates were used to replicate pre‐filtered velocity data provided by the proprietary software (OpenField v3.3.1, Catapult Sports, Melbourne, Australia). To assist the reader, we also describe the relative intensity of each player movement characteristic by expressing them per 1 minute of active duration.

For each movement characteristic described above, MIPs were calculated (defined as the maximum moving sum for the specified MIP duration within each discrete player‐match). This moving sum was calculated for continuous time‐series data, meaning that MIPs were inclusive of nonactive playing time (if applicable). Initially, we calculated MIPs for time windows ranging from 5 s to 10 min in increments of 5 s. Data from all increments were visualised, but preliminary analysis showed little difference for MIP durations longer than 5 min, so, for the sake of concision, we estimated statistical summaries at MIP durations likely to be useful for practitioners (5 s, 15 s, 30 s, 1 min, 2 min, 3 min, 4 min and 5 min). Further, MIP analysis was only conducted for total distance, high‐speed distance and the number of accelerations, decelerations and high‐speed cuts. Sprints were not considered for MIP analysis because they occurred rarely (∼2 per player, per match) and only accounted for ∼1% of the total distance. High‐intensity efforts were not considered for MIP analysis because they were highly associated with number and timing of accelerations. We were also interested if the 1 min MIPs for each of the movement characteristics occurred during the same time as the 1 min MIP for total distance (MIP_Distance_). To achieve this, for each player‐match, we extracted the value for each movement characteristic that was performed during the 1 min MIPdistance (i.e., the minute in the match that the player covered the most distance acted as the reference minute for the other characteristics).

Data were processed and visualised using MATLAB (Mathworks, vR2023b), and statistical analysis was conducted in R statistics (v4.3.0). Random effects linear mixed models were used (*lme4 R* package; 4; Bates et al. 2015) to estimate the mean and 95% confidence intervals (CIs_95%_) for each movement characteristic per player‐match, with random intercepts fitted for players and matches. Event data (e.g., number of accelerations) were modelled assuming a Poisson distribution and summary statistics were back‐transformed for ease of interpretation. Variation between players and matches were described using a trimmed 90% range (from 5th to 95th percentile).

## 3 Results

Of the data obtained from 28 players across 25 matches, five files were unable to be processed. Players competed in a median of 16 (range: 3–24) of the matches. Subsequently, data extraction was performed on 393 of 398 (98.7%) files and included in the analysis. The average number of satellite signals was 14 ± 1, with a horizontal dilution of precision of 0.71 ± 0.06.

A summary of player movement characteristics is presented in Table 1. On average, players were active for 52 min and covered 6057 m per match, with 795 m of high‐speed distance (> 5 m·s^−1^), and only 62 m of sprint distance (> 7 m·s^−1^). Players performed a similar number of high‐intensity efforts (*n* = 42) as accelerations (*n* = 44) and decelerations (*n* = 47) per match, yet very few sprints (*n* = ∼2). On average, players performed 14 high‐speed cuts per match. For all movement characteristics, variation between players was greater than between matches.

**TABLE 1:** Summary of movement characteristics for 28 elite Australian male field hockey players over 25 international matches (*n* = 393 player‐matches).

Performance characteristic	Mean (95% CI)^a^	Relative intensity^a^	Variation between player and match [90% trimmed range (5th–95th percentiles)]^b^	
			Between player	Between match
Active duration (min)	52 (50–55)	—	23 (40–63)	12 (47–59)
Total distance (m)	6057 (5853 to 6261)	116 (112–120) m·min^−1^	1942 (4761 to 6703)	879 (5759 to 6637)
High‐speed distance > 5 m·s^−1^ (m)^c^	795 (723–868)	15.2 (13.8–16.6) m·min^−1^	670 (432–1103)	295 (687–982)
Sprint distance > 7 m·s^−1^ (m)^c^	62 (36–107)	1.2 (1.2–2.0) m·min^−1^	118 (25–142)	72 (47–118)
High‐intensity efforts (n)^c^	42 (39–46)	0.81 (0.75–0.88) n·min^−1^	39 (32–71)	13 (38–50)
Sprints (n)^c^	2.2 (1.8–2.7)	0.04 (0.03–0.05) n·min^−1^	3.8 (0.8–4.7)	2.0 (1.7–3.6)
Accelerations > 2.5 m·s^−2^ (n)^c^	44 (41–48)	0.85 (0.78–0.92) n·min^−1^	42 (34–76)	11 (39–54)
Decelerations < − 2.5 m·s^−2^ (n)^c^	47 (44–51)	0.91 (0.85–0.98) n·min^−1^	36 (36–72)	17 (40–57)
High‐speed cuts 45° and > 5 m.s^−1^ (n)^c^	14 (12–16)	0.27 (0.23–0.31) n·min^−1^	24 (7–31)	5 (13–18)

^a^ Estimates are provided for per player and per match.

^b^ Variation between players and matches were described using a trimmed 90% range (from 5th to 95th percentile). Some trimmed ranges do note equal the difference between the 5th and 95% percentiles due to rounding.

^c^ Generalised models assumed a Poisson distribution and estimates were back‐transformed to their original units; 95% CIs: 95% confidence intervals.

**FIGURE 1:**
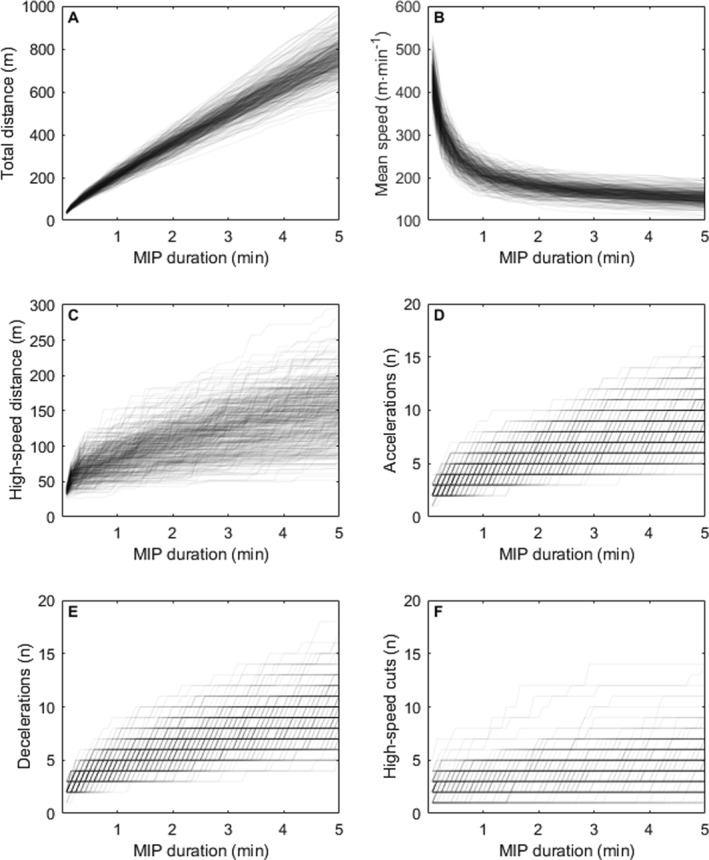
Maximum intensity periods (MIPs) from 5 s to 5 min for 28 elite Australian male field hockey players over 25 international matches. Different player movement characteristics are presented in the following panels: (A) total distance (m), (B) mean speed (m·s^−1^), (C) high‐speed distance (m), (D) accelerations (n), (E) decelerations (n) and (F) high‐speed cuts (n). Each semitransparent line illustrates MIPs of increasing durations for a single player‐match (*n* = 393), with darker areas indicating a higher density of player‐matches.

There was a nonlinear increase in the accumulation of distance covered (both total distance and high‐speed distance) and the number of accelerations, decelerations and high‐speed cuts with increasing MIP duration (Table 2, Figure 1). Specifically, MIPs of shorter durations were more intense. For example, players covered a mean of 37 m over their most intense 5 s MIP (equating to a mean speed of 448 m·min^−1^) whereas covering a mean of 768 m over their most intense 5 min MIP (mean speed: 154 m·min^−1^). Once again, we observed greater variation between players than between matches for every variable at each MIP duration.

**TABLE 2:** Summary of maximum intensity periods (MIPs) for 5 movement characteristics ranging from 5 s to 5 min for 28 elite Australian male field hockey players over 25 international matches (*n* = 393 player‐matches).

Performance characteristic	Maximum intensity period							
	5 s	15 s	30 s	1 min	2 min	3 min	4 min	5 min
Mean (95% CI)^a^								
Total distance (m)	37 (27–48)	81 (70–91)	129 (118–140)	210 (200–221)	360 (350–371)	502 (492–513)	638 (627–648)	768 (757–779)
Mean speed (m·min^−1^)^d^	448 (322–575)	322 (280–364)	258 (237–279)	210 (200–221)	180 (175–185)	167 (164–171)	159 (157–162)	154 (151–156)
High‐speed distance > 5 m·s^−1^ (m)^c^	37 (30–43)	60 (53–67)	71 (64–78)	84 (77–91)	105 (98–112)	124 (117–131)	139 (132–146)	152 (145–159)
Accelerations > 2.5 m·s^−2^ (n)^c^	2.2 (2.0–2.4)	2.8 (2.6–3.0)	3.5 (3.2–3.8)	4.3 (4.0–4.6)	5.6 (5.2–6.0)	6.8 (6.3–7.3)	7.7 (7.2–8.2)	8.5 (7.9–9.1)
Decelerations < − 2.5 m·s^−2^ (n)^c^	2.1 (1.9–2.3)	2.9 (2.7–3.1)	3.6 (3.4–3.9)	4.5 (4.2–4.9)	6.0 (5.6–6.3)	7.2 (6.8–7.7)	8.2 (7.7–8.7)	9.1 (8.5–9.6)
High‐speed cuts 45° and > 5 m.s^−1^ (n)^c^	2.1 (1.9–2.3)	2.3 (2.1–2.6)	2.5 (2.2–2.8)	2.8 (2.5–3.1)	3.2 (2.9–3.6)	3.6 (3.3–4.0)	3.9 (3.5–4.3)	4.2 (3.8–4.6)
Between player variation [90% trimmed range (5th–95th percentiles)]^b^								
Total distance (m)	6 (33–39)	17 (69–85)	31 (106–137)	62 (171–233)	111 (298–409)	153 (418–571)	215 (523–738)	275 (620–896)
High‐speed distance > 5 m·s^−1^ (m)^c^	6 (33–39)	23 (46–69)	36 (48–85)	48 (57–105)	66 (69–135)	77 (81–158)	90 (86–176)	101 (97–198)
Accelerations > 2.5 m·s^−2^ (n)^c^	0.9 (1.8–2.7)	1.6 (2.4–4.0)	1.8 (3.0–4.8)	2.7 (3.6–6.3)	4.1 (4.2–8.3)	4.9 (5.4–10.3)	6.2 (5.8–12.1)	6.8 (6.5–13.3)
Decelerations < − 2.5 m·s^−2^ (n)^c^	0.5 (2.0–2.5)	1.2 (2.3–3.5)	1.6 (2.9–4.5)	2.4 (3.7–6.1)	3.7 (4.6–8.3)	4.2 (5.8–10.0)	5.1 (6.6–11.7)	6.0 (7.1–13.0)
High‐speed cuts 45° and > 5 m.s^−1^ (n)^c^	1.3 (1.6–2.9)	2.0 (1.7–3.7)	2.5 (1.7–4.2)	3.1 (1.8–4.9)	3.8 (2.0–5.9)	4.8 (2.2–6.9)	5.1 (2.3–7.4)	5.9 (2.3–8.2)
Between game variation [90% trimmed range (5th–95th percentiles)]^b^								
Total distance (m)	3 (34–38)	7 (76–83)	13 (121–135)	24 (198–222)	28 (346–374)	44 (482–525)	72 (598–670)	111 (704–815)
High‐speed distance > 5 m·s^−1^ (m)^c^	3 (34–38)	13 (53–67)	22 (60–82)	24 (72–95)	30 (89–118)	33 (106–139)	37 (117–154)	45 (130–174)
Accelerations > 2.5 m·s^−2^ (n)^c^	0.9 (2.0–2.9)	1.1 (2.5–3.6)	1.4 (3.0–4.4)	1.5 (3.7–5.2)	1.9 (4.8–6.7)	1.9 (5.8–7.7)	2.2 (6.5–8.8)	2.6 (7.2–9.8)
Decelerations < − 2.5 m·s^−2^ (n)^c^	0.4 (1.9–2.3)	0.7 (2.5–3.2)	0.9 (3.1–4.0)	1.1 (4.0–5.1)	1.8 (5.0–6.8)	1.6 (6.3–7.9)	1.9 (7.1–9.0)	2.0 (8.0–10.0)
High‐speed cuts 45° and > 5 m.s^−1^ (n)^c^	0.7 (1.8–2.5)	0.7 (2.0–2.7)	0.8 (2.1–2.9)	0.9 (2.4–3.3)	1.2 (2.7–3.9)	1.1 (3.1–4.2)	1.1 (3.4–4.5)	1.2 (3.5–4.7)

^a^ Estimates are provided for per player and per match.

^b^ Some trimmed ranges do note equal the difference between the 5th and 95% percentiles due to rounding.

^c^ Generalised models assumed a Poisson distribution and estimates were back‐transformed to their original units; 95% CI: 95% confidence intervals.

^d^ Mean speed was included for ease of interpretation and not analysed separately. Instead, mean speed was calculated as the total distance divided by MIP duration in minutes for each interpretation.s

Table 3 presents the 1 min MIP for total distance (MIP_Distance_) and the corresponding values for each additional movement characteristic during this same 1 min period. On average, players covered 210 m during their 1 min MIP_Distance_. Players also covered a 64 m of HSD during their 1 min MIP_Distance_, which was 76% of the 84 m players covered on average during their 1 min MIP_HSD_. For all other movement characteristics, the 1 min MIP_Distance_ represented less than 40% of the 1 min MIP for that characteristic, highlighting that the MIPs for these discrete movement characteristics occur during different stages of the match. For example, players performed ∼4 accelerations during their 1 min MIP for accelerations but only ∼1 acceleration during the 1 min MIP_Distance_.

**TABLE 3:** Mean (standard deviation) of the 1 min maximum intensity period (MIP) for total distance (MIP_Distance_) and the corresponding values for each additional movement characteristic during the same 1 min period, for 28 elite Australian male field hockey players over 25 international games (*n* = 393 player‐matches).

Performance characteristic*	Mean (SD) during 1 min MIP_Distance_	Mean (SD) during 1 min MIP	1 min MIP ÷ 1 min MIP_Distance_ (%)
Total distance (m)	210 (19)	210 (19)	100%
High‐speed distance > 5 m·s^−1^ (m)	64 (29)	84 (21)	76%
Sprint distance > 7 m·s^−1^ (m)	9 (14)	28 (14)	32%
High‐intensity efforts (n)	1.2 (1.0)	4.0 (1.1)	30%
Sprints (n)	0.2 (0.4)	0.9 (0.4)	23%
Accelerations > 2.5 m·s^−2^ (n)	1.4 (1.3)	4.3 (1.3)	32%
Decelerations < − 2.5 m·s^−2^ (n)	1.7 (1.3)	4.5 (1.0)	38%
High speed cuts 45° & > 5 m.s^−1^ (n)	1.0 (1.1)	2.8 (1.1)	35%

^*^ Estimates are provided for per player and per match and standard deviations are calculated across *n* = 393 player‐matches.

**FIGURE 2:**
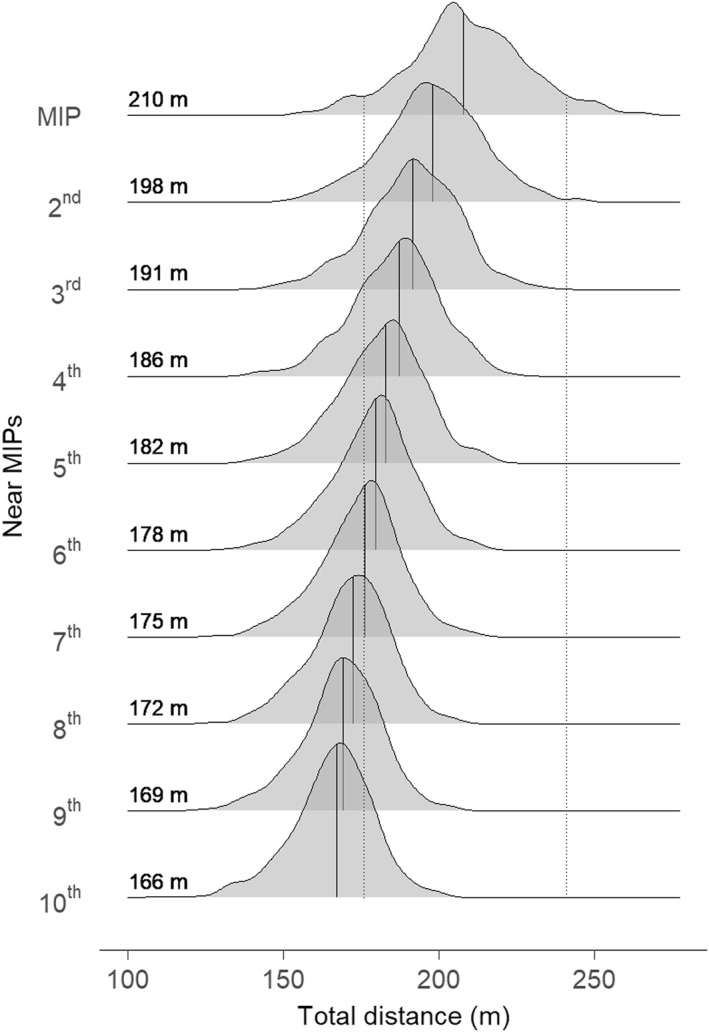
Distribution of the 10 most intense discrete 1 min periods for total distance performed by 28 elite Australian male field hockey players over 25 international matches (*n* = 393 player‐matches). Mean values are provided to the left of each distribution and illustrated by a solid vertical line, with dotted lines to represent the trimmed range (5th–95th percentile) of the maximum intensity period (MIP).

Finally, to visualise how often player movement demands reach near MIP intensities, Figure 2 illustrates the distribution of 1 min MIP_Distance_ as well as the nine next highest discrete periods (near MIPs) for total distance. There was considerable overlap observed between the distribution for 1 min MIP_Distance_ (210 m mean distance) and near MIPs. For example, of the 10th most intense 1 min period for each player‐match (166 m mean distance), nearly a quarter (22%) fell within the trimmed range of the MIP.

## 4 Discussion

The primary aim of this investigation was to examine the MIPs of various speed, acceleration‐based and change of direction variables during international male field hockey. We found that mean speed was ∼80% higher during the 1 min MIP than the match average (and still > 30% higher for the 5 min MIP). Even greater differences were observed in high‐intensity variables, with high‐speed distance, accelerations and decelerations accumulating > 5x faster during the 1 min MIP for those variables than the match average and high‐speed cuts occurring with ∼10x greater frequency. Our secondary aims included identifying variables that occurred too infrequently to warrant MIP analysis, with sprints (> 7 m·s^−1^) being determined to fit this criterion (62 m accumulated sprint distance over ∼2 sprints per match). We also found that players regularly reached high mean speeds throughout the match, with considerable overlap observed between the 1 min MIP_Distance_ and near MIPs. Finally, we determined that during the 1 min MIP_Distance_, all other movement characteristics were less than 40% of the 1 min MIP for that variable (except high‐speed distance: 76%), highlighting that the MIPs for these discrete movement characteristics occur during different stages of the match.

Mean speeds during international male field hockey have previously been reported as 116–127 m·min^−1^ across a whole match (James, Gibson, et al. 2021; Lythe and Kilding 2011; Polglaze et al. 2018), which is supported by our observations here (116 m·min^−1^). Therefore, it is noteworthy that we observed the 1 min MIP for mean speed (210 m·min^−1^) to be substantially greater than the only other previous investigation of MIPs in international male field hockey (157 m·min^−1^; Cunniffe et al. 2021). However, only a 3 min MIP was analysed in that investigation, with the authors cautioning that match average data could result in a misrepresentation of peak demands by up to 20%. Our data highlight that the 1 min MIP is over 80% greater than the match average, and even the equivalent 3 min MIP in our data (167 m·min^−1^) were ∼44% greater than match average. Beyond the magnitude of the peak periods identified here, our secondary analysis also highlighted that even the 10th most intense minute (166 m·min^−1^) was higher than the 3 min MIP reported in the only other international male field hockey investigation (Cunniffe et al. 2021). This highlights that the need to repeatedly run at intensities which are substantially higher than match averages occurs frequently during elite male field hockey matches. Due to the paucity of information on peak periods during field hockey, whether these movement characteristics are unique to international‐level players is currently not known. However, a recent investigation of national‐level male field hockey players found that they were unable to sustain their running performance in professional club matches, reporting a significantly decline in running performance from quarter 1 to quarter 2, and then again in quarter 4 (Lin et al. 2023). This provides some indication that the ability to maintain physical output throughout a match may be more developed in international players but requires further investigation.

In addition to having reported a greater mean speed during MIPs than previously observed, and highlighting that near maximal periods occur frequently throughout a match, we have also examined the MIPs for a more comprehensive range of movement characteristics (i.e., accelerations, decelerations and changes of direction) than previously investigated in international male field hockey. Previously, neither investigation of MIPs in international field hockey examined change of direction or acceleration‐based variables (Cunniffe et al. 2021; McGuinness et al. 2022), whereas Delves et al. (2021) and Büchel et al. (2023) both reported mean acceleration load across a range of MIP bands in club level athletes competing in a national field hockey competition. Importantly, it has been established that international field hockey athletes perform better in field‐hockey specific acceleration and change of direction tasks than national‐ and club‐level athletes (Tapsell et al. 2022). Additionally, the ability to perform repeated high‐speed change of direction tasks is only weakly associated with intermittent endurance performance in elite field hockey players (Goods et al. 2022). So, although mean acceleration load represents the overall change of speed demands, it conceals granular information that is increasingly being used to prescribe and monitor movement demands in elite team sport environments, such as discrete accelerations, decelerations and changes of direction (Goods et al. 2024; Riboli et al. 2024). In the present study, we found high‐speed distance, accelerations and decelerations during the 1 min MIP for those variables to occur at a rate > 5x more than the match average. This is similar to data recently reported in top‐class soccer, with high‐speed running (> 3.5x), accelerations (> 12x) and decelerations (> 7x) found in higher densities during MIPs than the equivalent difference for mean speed (+63%) (Riboli et al. 2024). Given the recent suggestion that MIPs are best used to inform the training intensity of team sport‐specific drills, such as small‐sided games (Weaving et al. 2022), and the knowledge that small‐sided games elicit lower mean speeds than competition in elite field hockey by design (Polglaze et al. 2015), the investigation of granular high‐intensity movement demands are warranted.

The decoupling of the MIP for mean speed and the MIP for other high‐intensity movements (accelerations, decelerations and high‐speed cuts) has not been investigated previously in field hockey, and thus, represents new information for practitioners using MIP data to inform training. Recent research in academy‐level soccer suggests that MIPs for mean speed are associated with high mean acceleration loads and heart rates, whereas high‐speed running and sprinting occur at different stages of the match (Kim et al. 2023). Our data suggest that the MIPs for discrete acceleration, deceleration and change of direction events occur during different stages of the match to the 1 min MIP_Distance_, reaching less than 40% of the 1 min MIP for those variables. Whether these intense periods for different movement demands are occurring during the same stage of the match (i.e., same rotation, quarter or half) warrant further investigation. This supports our earlier suggestion that practitioners should consider monitoring the frequency of high‐intensity movements during field hockey‐specific training drills rather than mean speed, which does not occur concurrently during matches, and is known to be lower during small‐sided games in elite field hockey (Polglaze et al. 2015). If high‐intensity movements are monitored in small‐sided games as an index of MIP during matches, practitioners can also reasonably expect that players will improve aerobic endurance performance similarly to conventional endurance training, making this an efficient training option for simultaneously achieving technical, tactical and physical goals (Moran et al. 2019).

There are several limitations worth highlighting in the current investigation. Although the analytical approach taken here is the most robust to date in field hockey, it is a representation of a single national team across a ∼13‐month period; so, it is possible that contextual factors, such as style of play, might influence the results. Additionally, intra‐match contextual factors, such as opponent ranking (James et al. 2023), whether attacking or defending (Cunniffe et al. 2022), or environmental conditions (Goods, Maloney, et al. 2023; James, Willmott, et al. 2021) were not available for these data and may influence match movement demands. However, due to the quality of the team and opposition in this analysis as well as the number of observations, we are confident that the data presented here provide useful insight into the MIPs of international male field hockey. Regardless, future replication studies of other international teams (both male and female) are warranted to confirm the data currently available. Additionally, in line with our secondary aims, we encourage researchers in other team sports to also consider reporting a broad range of movement characteristics, including near peak periods during the match, and whether they occur simultaneously during matches.

## 5 Conclusion

Mean speed during the most intense minute of international male field hockey is ∼80% higher than the match average. Further, the 10th most intense minute during a match remains ∼44% higher than match average, which is similar to the 3 min MIP observed here, and higher than any previously reported MIP for international male field hockey. High‐speed distance, accelerations and decelerations accumulate > 5x faster during the 1 min MIP for those variables than the match average, which increases to ∼10x faster for high‐speed cuts. Finally, during the 1 min MIP_Distance_, players performed 76% of their 1 min MIP for high‐speed distance, but for all other movement characteristics, this dropped to less than 40% of the 1 min MIP for that variable. This highlights that the MIPs for these discrete movement characteristics occur during different stages of the match.

## Ethics Statement

Institutional ethical approval was obtained for this project at the University of Western Australia Human Research Ethics Committee (2021/ET000838).

## Conflicts of Interest

The authors declare no conflicts of interest.

## Data Availability

The authors declare that this manuscript has not been published or submitted elsewhere and that it will not be submitted for publication elsewhere until a final decision has been made as to its acceptability by the Journal and also declare that no material has been reproduced from other sources.
